# Supplementary material for: Transcriptional super-enhancers control cancer stemness and metastasis genes in squamous cell carcinoma
Source: Nat Commun. 2021 Jun 25;12:3974. doi: 10.1038/s41467-021-24137-1 (PMC8233332; doi:10.1038/s41467-021-24137-1)
Supplement: Supplementary file 1 — Supplementary Information [file 41467_2021_24137_MOESM1_ESM.pdf]

Supplementary Information for

Transcriptional Super-Enhancers Control Cancer Stemness and Metastasis Genes in Squamous Cell Carcinoma

Jiaqiang Dong[1,2,6], Jiong Li[3,4,6*], Yang Li[1,2], Zhikun Ma[3,4], Yongxin Yu[1,2], and Cun-Yu Wang[1,2,5*]

1. Jonsson Comprehensive Cancer Center and Broad Stem Cell Research Center, UCLA, Los Angeles, CA 90095, USA.
2. Laboratory of Molecular Signaling, Division of Oral Biology and Medicine, School of Dentistry, UCLA, Los Angeles, CA 90095, USA.
3. Department of Medicinal Chemistry, School of Pharmacy, Virginia Commonwealth University, Richmond, Virginia 23284, USA.
4. Department of Oral and Craniofacial Molecular Biology, School of Dentistry, Richmond, Virginia 23284, USA.
5. Department of Bioengineering, Henry Samueli School of Engineering and Applied Science, UCLA, Los Angeles, CA 90095, USA.
6. These authors contributed equally

*Correspondence and requests for materials should be addressed to C.-Y.W. (cunywang@ucla.edu) or J.L. (jli29@vcu.edu)

This PDF file includes:

Supplementary Tables

Supplementary Figures and Legends

Supplementary Table 1. The primers used for RT-qPCR.

Primer Name	Directions	Sequence
GAPDH	Forward	TCATTGACCTCAACTACATG
	Reverse	TCGCTCCTGGAAGATGGTGAT
MMP3	Forward	CTGGACTCCGACACTCTGGA
	Reverse	CAGGAAAGGTTCTGAAGTGACC
IL1A	Forward	CAATTTTAGGAGGACCAGAGCTAC
	Reverse	CTGTGGCTTCTTGTTTCAACTGCT
IL1B	Forward	AGGAGAATGACCTGAGCACCTTC
	Reverse	CGTGCACATAAGCCTCGTTATCC
IL6	Forward	CCTGAACCTTCCAAAGATGGC
	Reverse	TTCACCAGGCAAGTCTCCTCA
IL8	Forward	ACTGAGAGTGATTGAGAGTGGAC
	Reverse	AACCCTCTGCACCCAGTTTTC
CXCL1	Forward	CAGGGAATTCACCCCAAGAACATCC
	Reverse	TTTCCGCCCATTCTTGAGTGTGG
TNFAIP3	Forward	TCAACTGGTGTCGAGAAGTCC
	Reverse	CAAGTCTGTGTCCTGAACGC
BCL2	Forward	GGTGGGGTCATGTGTGTGGA
	Reverse	CGGTTCAGGTACTCAGTCATCC
BCL3	Forward	AACCTGCCTACACCCCTATAC
	Reverse	CACCACAGCAATATGGAGAGG
BIRC3	Forward	AAGTGGTTTCCAAGGTGTGAGTAC
	Reverse	CAGCTGTTCAAGTAGATGAGGGT
MET	Forward	GCCTCCTTCTGGGAGACATCATAGT
	Reverse	TCGATGGCCTTTTAAAGGTCAGG
FOSL1	Forward	AACTGGAAGATGAGAAATCTGGGC
	Reverse	CTCCTTCCGGGATTTTGCAGATG
CD44	Forward	CCGGGAGGGCTGCTACTTCTTAAA
	Reverse	GGCTGCAGTTTTTATTCGAGGTTG
BMI1	Forward	TGAAGATAGAGGAGAGGTTGCAG
	Reverse	TTCCGATCCAATCTGTTCTGGTC
AURKB	Forward	CAGAAGAGCTGCACATTTGAC
	Reverse	CCTTGAGCCCTAAGAGCAGATT
BANF1	Forward	TGGCTGAAAGACACTTGTGG
	Reverse	CACTCTCGAAGGCATCCGAAG
BUB1B	Forward	GCACCGACAATTCCAAGCTC
	Reverse	TGTGCTTCGTTGTGGTACAGA
CCND2	Forward	GACAGCATCACGCTGCATCC
	Reverse	CAGGGCACAGTCTGGAGAGG
CDC20	Forward	GCTTTGAACCTGAACGGTTTTG

	Reverse	TCTGGCGCATTTTGTGGTTTT
CHAF1A	Forward	CAGCCAGACAGTCTTGTGGAC
	Reverse	GTCGTTCTGAATGGCCTTCAA
KIF22	Forward	GCTTCGGGAGACCTGGTAATC
	Reverse	AGTGCCGCTCAAAATCAGCA
MCM5	Forward	GGAAGTGCAACACAGATCAGG
	Reverse	AGGGACGACCTTGTCACACA
NCAPD2	Forward	AAACGCCCATCTAAATGCCCT
	Reverse	TCCGAGCTTTCTTACCCTTCC
YAP1	Forward	TCCCAGACTACCTTGAAGCC
	Reverse	GCATCAGCTCCTCTCCTTCT
TP63	Forward	CTGAACTGAAGAAACTCTACTGCC
	Reverse	GCTCAGCTTTTTTGTAGACAGGC

Supplementary Table 2. The primers used for ChIP-qPCR.

Primer Name	Directions	Sequence
TP63 SE	Forward	GGCAGGTGTTATTCTCACTTCTCAC
	Reverse	GAAGGCTCATTCCCCTACTTTCCAA
TP63 NEG	Forward	ACAAACGAGAGATCCACAGGTTCAG
	Reverse	GCTCATTGATGCCATGGTTTCTGAG
MET SE	Forward	GGTTCCTGGGCACCGAAAGGTAAA
	Reverse	GGGGAACAAAGAAAGCAGAACCC
MET NEG	Forward	CCCTGAGAATGATCAGCAAA
	Reverse	GTCGCAGATGAGGTAAAGCA
BIRC3 SE	Forward	GAGTTCCCCTAAGTCCTAAAAGG
	Reverse	AAATCCCCACCCCTATCTGTACC
BIRC3 NEG	Forward	CCTGGTGCAGAGCTGAGTTCACTT
	Reverse	TACAGGAGCACCCAGATCCATAA
MMP3 SE	Forward	AGTCCTCCTATCTCAGTCTCCCA
	Reverse	CTAGCCAAGGAAAGAATCCCCTAT
MMP3 NEG	Forward	TCAAGCTGGATGATCATTTCTGGC
	Reverse	GGCAGAGTGAGACTCCGTTTCAA
CXCL1 Promoter	Forward	ACAGCCTCGCTCAGTCAGTGAGTCTCTT
	Reverse	GGGACTTCACGTTCACACTTTGGATGTT
CXCL1 NEG	Forward	ATGTATACATGTGCCATGCTGG
	Reverse	GTTATTCAGAAACAACCTTGGA
ICAM1 Promoter	Forward	CCTCTGCTACTCAGAGTTGCAACCTC
	Reverse	AACAGAGCCCCGAGCAGGACCA
ICAM1 NEG	Forward	CCTTTGCCAACCTTCCCATTCT
	Reverse	TGAGTTGCCCCTGGGAAAGATC
LTB Enhancer	Forward	AAGGACCTCCAAGACCTGAATA
	Reverse	GAAACCGAGCACTGGAATCATG
LTB NEG	Forward	GCCCAGCCCCAGTATTCAGTTT
	Reverse	CAGAGTCAGTGGCAGAACCAGA

Supplementary Table 3. The gRNA sequences used for cloning.

gRNA	Sequence
MMP3	AATTCTAAGCCGCTGATCCC
TP63	GGAATGCTACCTTCTAGCAT

Supplementary Table 4. The primers used for cloning.

Primer Name	Directions	Sequence
TP63 SE	Forward	CAAGCTTTTGGCTCACGCGTCAGACTTCCTAT
	Reverse	GCTCGAGGTAAAAGCAATGAAATTTTTCAGCA
TP63 NEG	Forward	CAAGCTTCAAACAAACGAGAGATCCACAGGTTC
	Reverse	GCTCGAGTCCTTCTGCCTCAGCTTCCTGTGTA

Supplementary Figure 1

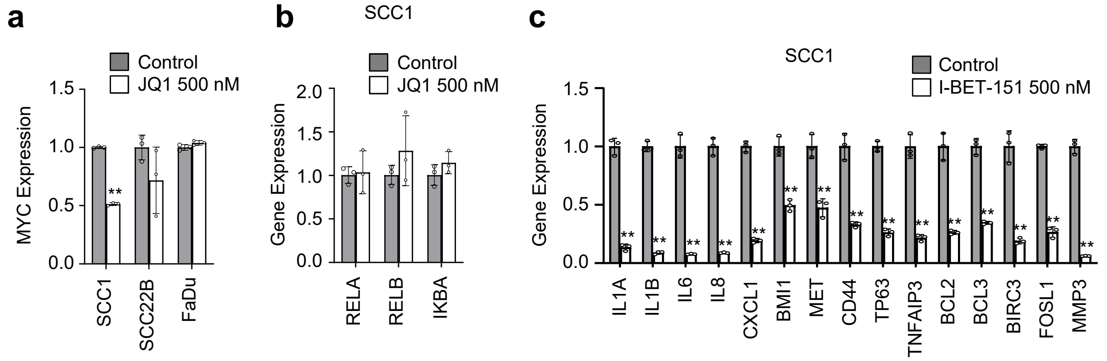

Supplementary Figure 1. BET inhibitors inhibit the expression of cancer stemness genes and pro-invasive genes in SCC cells. a, JQ1 had mild effect in suppression of *MYC* expression in SCC cells (n=3 per group). **b,** JQ1 did not inhibit *RELA*, *RELB*, and *IKBA* expression in SCC1 cells (n=3 per group). **c,** I-BET-151 inhibited the expression of cancer stemness genes and pro-invasive genes in SCC cells (n=3 per group). Data are presented as mean values ± SD in **a**, **b**, and **c**. Statistical analysis was performed using two-tailed unpaired Student's-*t* test. $^{**}P<0.01$. The data in **a**-**c** are representative of 3 experiments with similar results. Source data are provided as a Source data file. The precise *P* values are summarized in Source data file.

Supplementary Figure 2

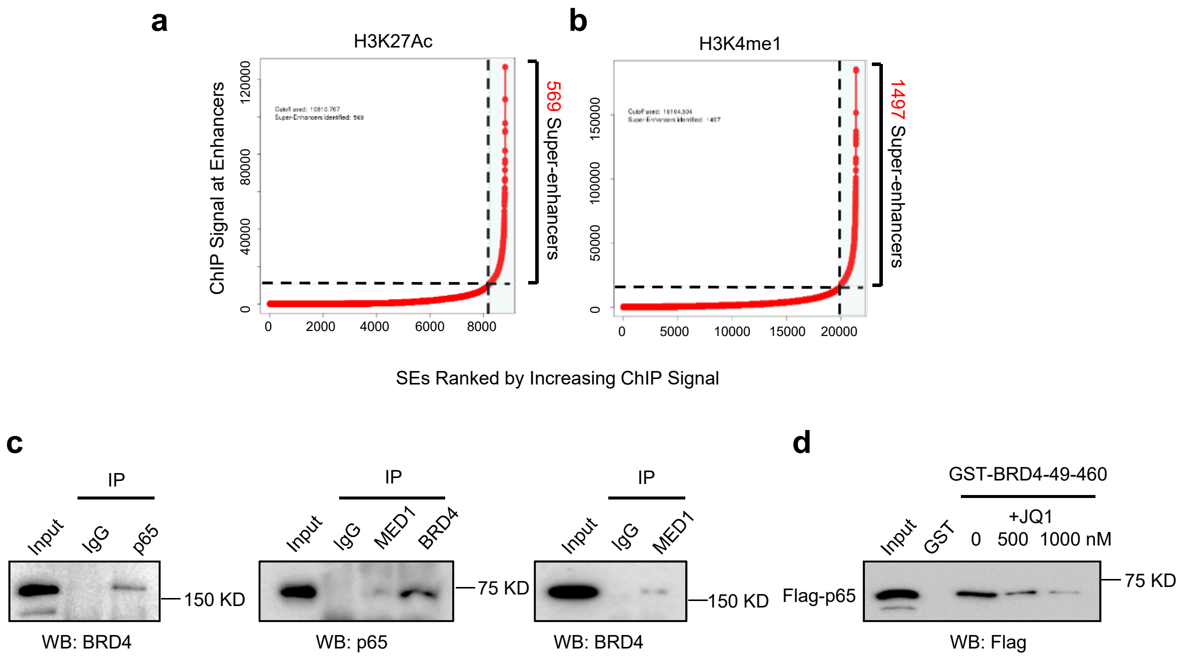

Supplementary Figure 2. SEs control the expression of cancer stemness genes and pro-invasive genes. a, Identification of SEs by ChIP-seq of H3K27Ac. **b**, Identification of SEs by ChIP-seq of H3K3me1. **c,** BRD4, MED1, and p65 interacted endogenously in SCC1 cells. **d,** JQ1 inhibited the interaction between BRD4 and p65. The data in are representative of 3 experiments with similar results for **c** and **d**. Source data are provided as a Source data file.

Supplementary Figure 3

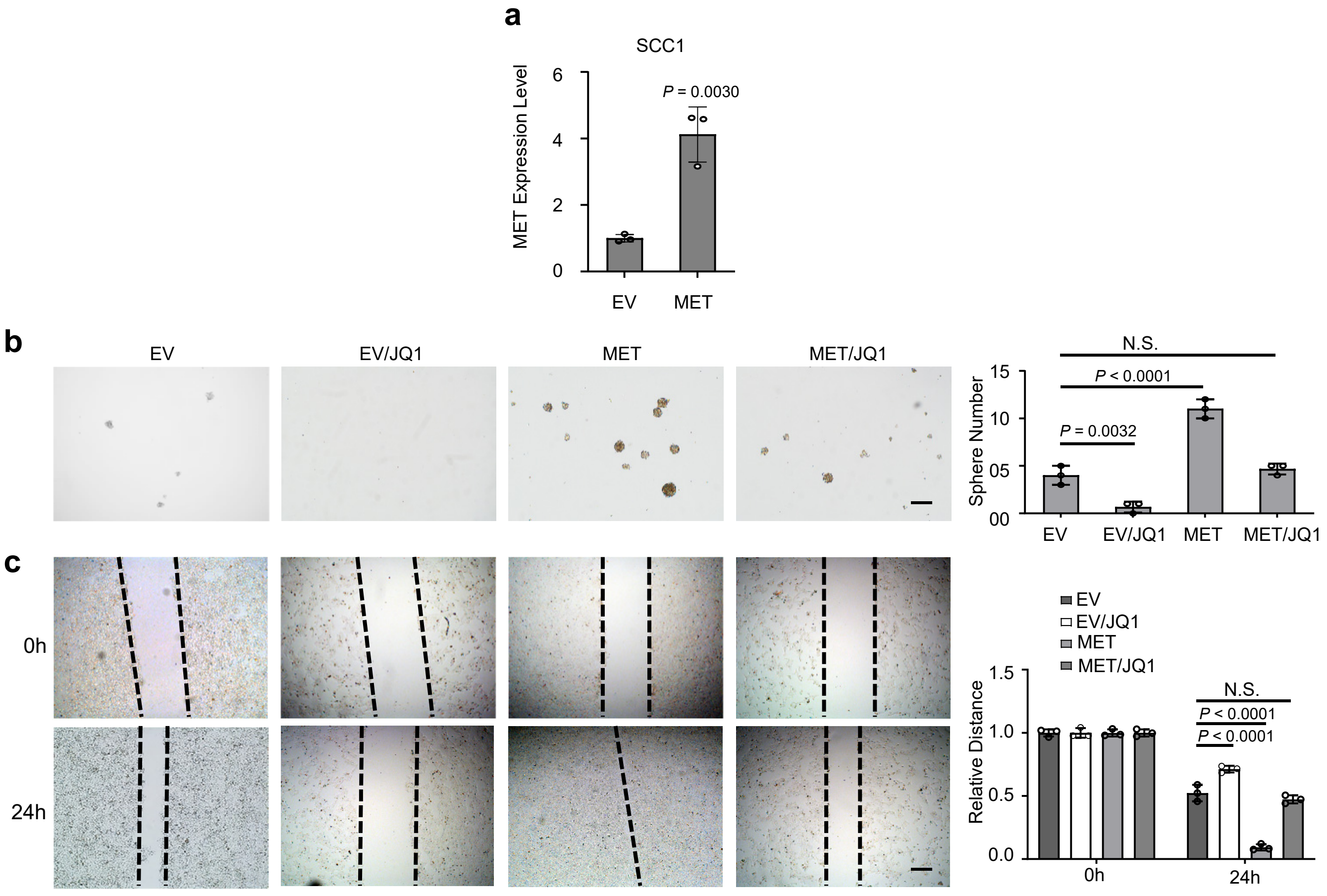

Supplementary Figure 3. Overexpression of MET rescues the malignant phenotypes of SCC cells suppressed by JQ1. a, Overexpression of *MET* in SCC1 cells by qRT-PCR (n=3 per group). **b**, Overexpression of MET in SCC1 cells attenuated the inhibition of tumorsphere formation by JQ1 (n=3 per group). Scale bar, 200 μm. **c**, Overexpression of MET in SCC1 cells attenuated the inhibition of migration growth by JQ1 (n=3 per group). Scale bar, 200 μm. Data are presented as mean values ± SD in **a**, **b**, and **c**. Statistical analysis was performed using two-tailed unpaired Student's-*t* test in **a**. Statistical analysis was performed using two-way ANOVA in **b** and **c**. N.S., not significant; EV, SCC1 cells expressing empty vector; MET, SCC1 cells overexpressing *MET*. The data in **a**-**c** are representative of 3 experiments with similar results. Source data are provided as a Source data file.

Supplementary Figure 4

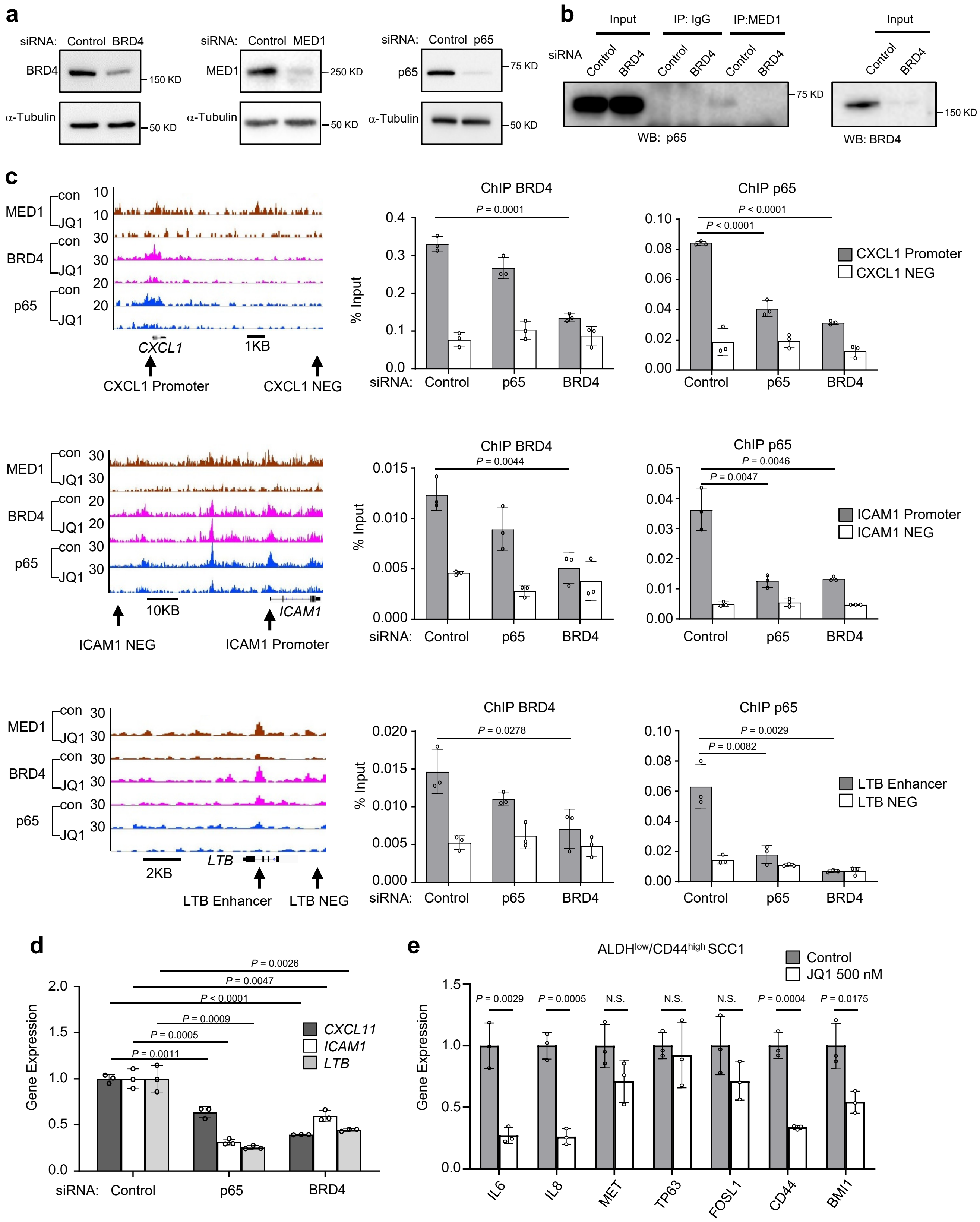

Supplementary Figure 4. BRD4 recruits MED1 and p65 to form SEs. a, Knockdown of BRD4, MED1, and p65 by siRNA. **b,** Knockdown of BRD4 inhibited the interaction between MED1 and p65 in SCC1 cells. **c,** Knockdown of BRD4 impaired p65 recruitment to the promoters or enhancer regions of *CXCL1*, *ICAM1*, and *LTB* (n=3 per group). **d,** Knockdown of BRD4 or p65 significantly inhibited the expression of *CXCL1*, *ICAM1*, and *LTB* in SCC1 cells (n=3 per gorup). **e,** RT-qPCR showed that JQ1 had little effect in suppression of SE-associated gene, *TP63*, *MET*, and *FOSL1* expression in non-CSCs from SCC1 (n=3 per group). Data are presented as mean values ± SD in **c**, **d**, and **e**. Statistical analysis was performed using two-tailed unpaired Student's-t test. The data in **a-e** are representative of 3 experiments with similar results. Source data are provided as a Source data file.

Supplementary Figure 5

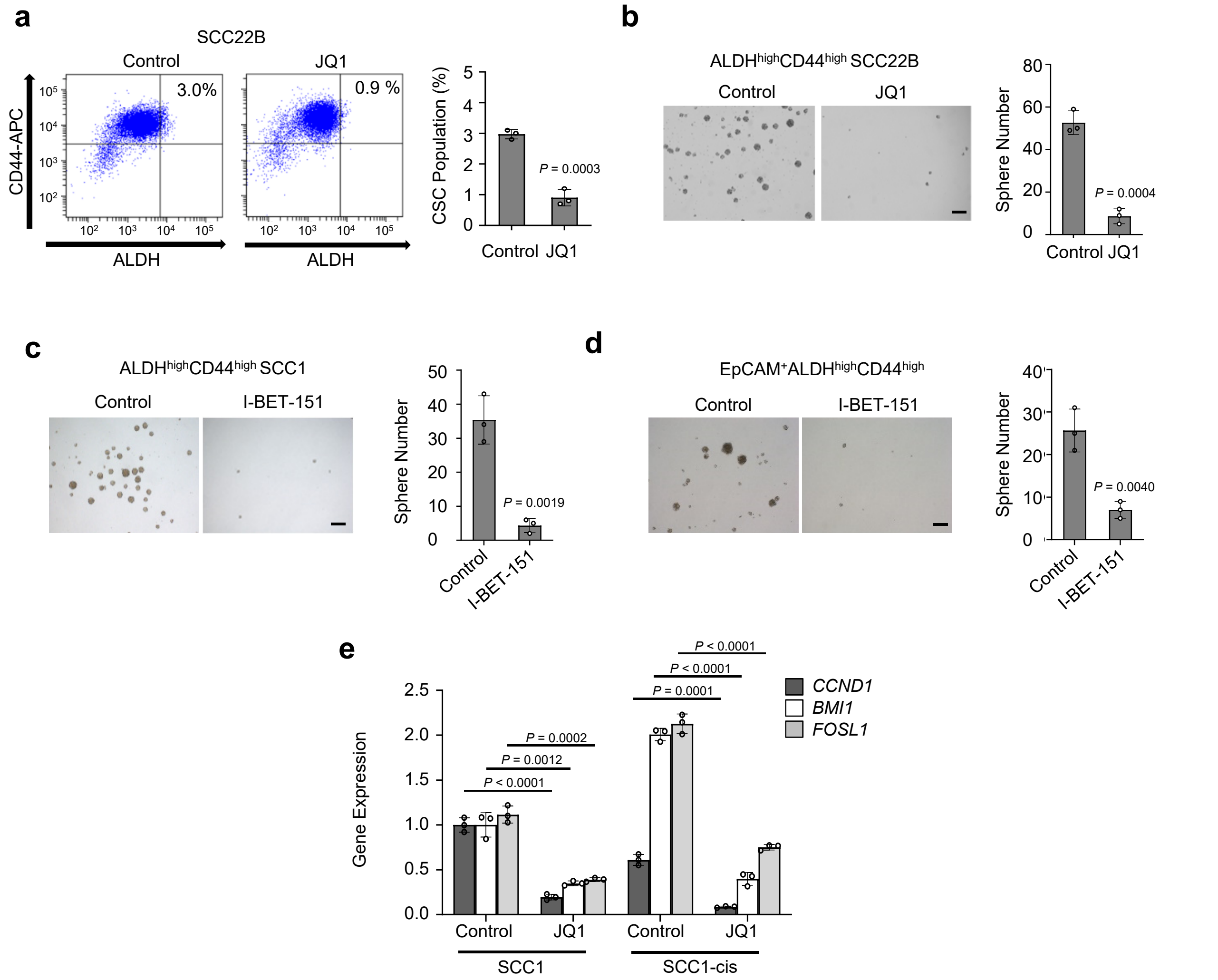

Supplementary Figure 5. BET inhibitors inhibit tumorsphere formation of CSCs from human HNSCC. a, JQ1 reduced the percentage of $CD44^{high}ALDH^{high}$ CSC-like cells in human SCC2B cells. Values are mean ± SD for triplicate experiments Statistical analysis was performed using two-tailed unpaired Student's-*t* test. **b**, JQ1 inhibited tumorsphere formation of $CD44^{high}ALDH^{high}$ cells from human SCC22B cells. Values are mean ± SD for triplicate experiments. Statistical analysis was performed using two-tailed unpaired Student's-*t* test. Scale bar, 200 μm. **c,** I-BET-151 inhibited tumorsphere formation of $CD44^{high}ALDH^{high}$ cells from human SCC1 cells. Values are mean ± SD for triplicate experiments. Statistical analysis was performed using two-tailed unpaired Student's-*t* test. Scale bar, 200 μm. **d,** I-BET-151 inhibited tumorsphere formation of $EpCAM^{+}CD44^{high}ALDH^{high}$ CSCs from human PDXs of HNSCC. Values are mean ± SD for triplicate experiments Statistical analysis was performed using two-tailed unpaired Student's-*t* test. Scale bar, 200 μm. **e,** JQ1 inhibited *CCND1*, *BMI1*, and *FOSL1* expression in SCC1 and SCC1-cis cells (n=3 per group). Data are presented as mean values ± SD. Statistical analysis was performed using two-tailed unpaired Student's-*t* test. The data in **e** are representative of 3 experiments with similar results. Source data are provided as a Source data file.

Supplementary Figure 6

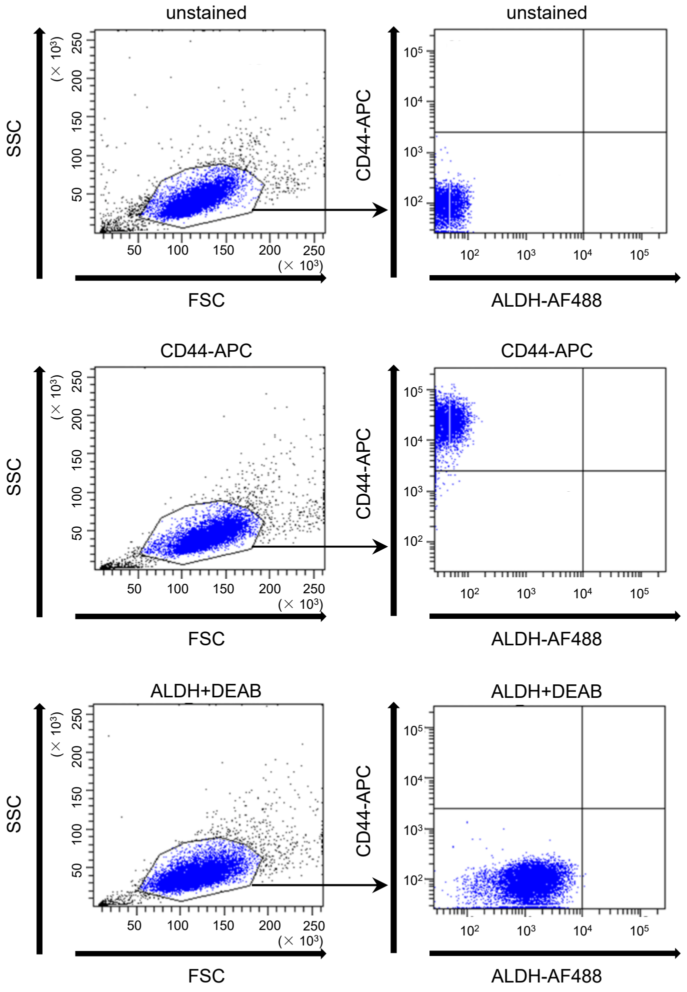

Supplementary Figure 6. Gating strategy to determine CD44^{high}ALDH^{high} CSC-like cells in human SCC. Related to Figs. 6a, 6d, 6g, and Supplementary Fig. 5c. The gate was determined based on "ALDH+DEAB" sample. The negative control and single CD44-APC stained sample were used as data quality control. FSC, forward scatter; SSC, side scatter; APC, Allophycocyanin; AF488, Alexa Fluor 488; DEAB, N,N-diethylaminobenzaldehyde.
