# Supplementary material for: Transcriptional super-enhancers control cancer stemness and metastasis genes in squamous cell carcinoma
Source: Nat Commun. 2021 Jun 25;12:3974. doi: 10.1038/s41467-021-24137-1 (PMC8233332; doi:10.1038/s41467-021-24137-1)
Supplement: Supplementary file 3 — Description of Additional Supplementary Files [file 41467_2021_24137_MOESM3_ESM.pdf]

Description of Additional Supplementary Files

Supplementary Data 1. Differentially expressed genes in FaDu, SCC22B and SCC1 cells treated with JQ1.
